# Les perdus de vue en radiothérapie: expérience de l'Institut National d'Oncologie au Maroc

**DOI:** 10.11604/pamj.2014.19.18.4445

**Published:** 2014-09-08

**Authors:** Imane Mezouri, Hanane Chenna, Sara Bellefqih, Hanan Elkacemi, Tayeb Kebdani, Noureddine Benjaafar

**Affiliations:** 1 Service de Radiothérapie, Institut National d'Oncologie, Université Mohammed V Souissi, Rabat, Maroc

**Keywords:** Perdu de vue, radiothérapie, cancer, traitement, INO, lost to follow-up, radiotherapy, cancer, treatment, INO

## Abstract

Les perdus de vue (PDV) sont toute personne incluse dans un programme et dont on est sans nouvelles depuis six mois. L'objectif de cette étude est de fournir une description objective du problème des malades PDV au service de radiothérapie à l'Institut National d'Oncologie (INO), elle permet d’étudier l'impact des facteurs socio-économiques, démographiques et ceux liés à la maladie entraînant l'abandon du traitement par le patient. Nous avons réalisé une étude rétrospective de 77 patients PDV parmi 2254 patient admis à l'INO du premier janvier au 31 décembre 2011 pour traitement par radiothérapie. La présente analyse a mis en évidence que les taux d'abandon sont associés à des facteurs liés à la maladie et qu’à la fois le patient et le médecin doivent être formés et être conscients de la façon dont les stades avancés de la maladie, le mauvais statut de performance ainsi que la combinaison des autres problèmes de santé peuvent suffisamment conduire le patient à l'abandon du traitement.

## Introduction

Dans la majeure partie des articles est perdu de vue (PDV) toute personne incluse dans un programme et dont on est sans nouvelles depuis six mois [1]. A noter qu'il y a une hétérogénéité des définitions. On distingue trois situations: (1) Les morts; (2) Les personnes vivantes mais ne venant plus aux centres de prise en charge; (3) Les patients suivis dans un autre centre. Il y a une autre définition différente: c'est la notion d'attrition (rupture de traitement) qui inclut les décès, les perdus de vue, les patients suivis mais non observant mais exclus les patients suivis dans un centre différent. En raison de l'existence des difficultés d'accès aux soins dans de nombreux pays en développement, on trouve qu'il y a une proportion assez importante des malades qui ne terminent pas le traitement qui leur était prescrit. Le but de ce travail est de fournir une description objective du problème des malades perdus de vue au service de radiothérapie à l'Institut National d'Oncologie (INO). La présente étude a donc été menée pour étudier l'impact des facteurs socio-économiques, démographiques et ceux liés à la maladie entraînant l'arrêt et l'abandon du traitement par le patient afin de permettre l'optimisation de la prise en charge des patients en radiothérapie, d'adapter le fractionnement et l’étalement en fonction de l’état général du patient et de prendre en compte ses conditions socio-économiques.

## Méthodes

Durant l'année 2011, 2254 patients ont été vus en consultation de radiothérapie et chez qui l'indication d'une irradiation a été retenue: 556 patients (25%) ont eu une simulation conventionnelle et 1698 patients (75%) ont bénéficié d'un Scanner dosimétrique avant de commencer le traitement. Parmi ceux-ci, 77 patients ont été perdus de vue soit avant la première séance de la radiothérapie soit en cours du traitement.

Nous avons réalisé une étude rétrospective de 77 patients admis à l'INO du premier janvier au 31 décembre 2011 pour traitement par radiothérapie. Pour la collecte des données, on a utilisé en plus des dossiers cliniques des patients, le système d'enregistrement et de vérification (MOSAIC). Les facteurs sociodémographiques et économiques inclus dans la présente analyse étaient l’âge, le sexe, l’état matrimonial, le niveau de l’éducation: analphabète, alphabète et inconnu, le revenu familial mensuel (le revenu familial mensuel a été évalué en fonction de divers facteurs tels que l'occupation du patient, le travail du conjoint..), le nombre d'enfants (<2,> 2, et inconnu ou sans enfants), et le lieu de la résidence. Les facteurs liés à la maladie inclus étaient la localisation de la tumeur, le stade de la maladie, le statut de performance de l'OMS (PS) et les co-morbidités (le diabète, l'hypertension artérielle (HTA), la broncho-pneumopathie chronique obstructive (BPCO), la cardiopathie ischémique et l'asthme).

## Résultats

### Caractéristiques des patients

L’âge des patients variait de 22 à 86 ans avec un âge moyen de 55 ans. 51malades (66.2%) habitent dans des villes situées à plus de 100 Km de notre centre (Rabat) et 26 patients habitaient à moins de 100 Km du centre de RTH. 65% des patients étaient mariés. 65% des patients avaient un faible revenu mensuel, 26% moyen et 3% haut avaient un haut niveau socio-économique. 58% des patients étaient analphabètes alors que seulement 1.5% de nos patients avaient un enseignement supérieur. 75% des patients avaient une profession dite ménagère, 3% étaient des fonctionnaires de l’état, 22% étaient des ouvriers d'usine. 68% des patients avaient plus de 2 enfants. 40% des malades étaient suivis pour un diabète non insulinodépendant (DNID), 10% étaient hypertendus sous traitement, 30% étaient porteurs d'une BPCO, 10% étaient connus asthmatiques. Tableau 1

**Tableau 1:** Caractéristiques des patients

Caractéristiques des patients	N (%)
**Age:** (Moyen 55 ans (22-86))	
< 50ans	22 (2.,7)
50-59 ans	25 (32,46)
60-69 ans	12 (15,58)
>70 ans	18 (23,38)
**Sexe**	
Féminin	35 (45,5)
Masculin	42 (54,5)
**Etats general**:	
PS: 0-1	20 (26)
PS: 2	27 (35)
PS 3	20 (26)
PS 4	8 (10)
**Comorbidités**	
DNID	30 (40)
HTA	9 (10)
DNID + HTA	11 (15)
BPCO/ASTHME	30 (40)
CARDIOPATHIE	3 (3)
**Statut matrimonial**	
Célibataire	17 (20)
Marié	50 (65)
Divorcé/veuf	10 (13)
**Nombre d'enfant**	
>2	52 (68)
1-2	9 (12)
0	16 (20)
**Revenu mensuel**	
Faible	50 (65)
Moyen	20 (26)
Haut	2 (3)
Non précisé (NP)	5 (6)
**Education**	
Analphabètes	45 (58)
Ecole primaires	17 (22,5)
Ecole secondaire	14 (18)
Enseignement supérieur	1 (1,5)
**Profession**	
Journaliers	58 (75)
Ouvriers	17 (22)
Profession	2 (3)

### Caractéristiques des tumeurs

Le siège de la tumeur était au niveau pulmonaire dans 20 cas dont 14 étaient stade IV, 15 malades avaient une tumeur du col utérin, les cancers de la sphère ORL ont été retrouvés chez 14 cas dont le stade IV a été retrouvé chez 10 patients. Le Tableau 2 résume la localisation ainsi que le stade des tumeurs.

**Tableau 2:** Caractéristiques des tumeurs

Siège des tumeurs	Nombre de cas: n (%)	Stades
Poumon	20 (26)	Stade IIIB: 6cas
		Stade IV: 14 cas
Col utérin	15 (19,5)	Stade IIB distal: 6 cas
		Stade IIIB: 7cas
		Stade IV: 2 cas
Sphère ORL	14 (18,1)	
Cavum	3	Stade III: 2 et stade IV: 1
Pharyngo-larynx	6	Stade III: 1 et stade IV: 5
Cavité buccale	3	Stade IV
Autres	2	Stade IV
Sein	10 (13)	2 Stade IIB: 2, stade III: 6 et stade IV: 2
Encéphale: glioblastome	4 (5,2)	
Rectum	4 (5,2)	Stade IIA: 2, stade III: 1 et NP: 1
prostate	3 (3,9)	2 Stade disséminé et 1 haut risque non métastatique
Estomac	2 (2,6)	Stade III
Utérus	2 (2,6)	Stade IV
Autres	3 (3,9)	NP

### Délai d'attente

Le délai moyen entre la première consultation à l'INO et la consultation en radiothérapie (RTH) était de 11 jours avec des extrêmes allant de zéro jour à 42 jours (surtout pour compléter le bilan d'extension de la maladie). Le délai moyen entre la consultation RTH et le centrage était de sept jours avec des extrêmes allant de zéro jour à 55 jours (le retard est dû au bilan d'extension qui n'est pas toujours complet). Le délai moyen entre le centrage et le début du traitement était de 13 jours avec des extrêmes allant de deux jours à 30 jours.

### **Le suivi des patients**: Tableau 3

En analysant ces résultats, on trouve que les conditions démographiques et socioculturelles ainsi que les facteurs liés au malade tels que l’âge avancé (plus de 60 ans), l’état matrimonial, le niveau des études, le faible revenu, l'augmentation du nombre des enfants, le lieu de résidence loin du centre du traitement étaient significativement associés à la perte de vue et l'abandon du traitement. Les facteurs importants liés à la maladie étaient le PS de l'OMS avant le traitement et le stade de la maladie au moment du diagnostic. Les patients âgés de plus de 60 ans étaient plus perdus de vue par rapport aux patients plus jeunes (<50 ans). Les patients veufs, divorcés ou séparés ou célibataires étaient plus susceptibles d’être PDV par rapport aux femmes mariées. Les patients ayant fait des études primaires étaient plus susceptibles d’être PDV par rapport aux patients ayant une étude secondaire ou supérieure. Les patients qui vivaient loin du centre étaient plus susceptibles d'abandonner leur traitement. Le mauvais état de la performance des patients avant le traitement a augmenté la probabilité d’être PDV. Les patients ayant des stades avancés étaient plus susceptibles d’être PDV. Le délai d'attente a été respecté et n'avait pas un impact négatif dans cette étude. Par contre, le bilan d'extension initial devait être complet avant de poser l'indication de l'irradiation et avant de faire les coupes scanner-dosimétriques. Le fractionnement et l’étalement ont dû être beaucoup plus brefs pour les malades altérés et dans les stades avancés de la maladie (le schéma hypofractionné: une seule séance de huit Gy ou deux séances de 6,5 Gy).

**Tableau 3:** Le suivi des patients

	Perdu de vue avant traitement (n = 46)		Perdu de vue en cours du traitement (n = 31)	
Causes	RTH Palliative n = 19	RTH Curative n = 27	RTH palliative n = 11	RTH curative n = 20
**Décès**: n = 55 (71, 4%)	Stade IV: 15	Stade IV: 5	Stade IV : 10	Stade IV : 2
	Stade III: 3	Stade III: 5		Stade III : 6
	Glioblastome: 1	Glioblastome:1		Prostate haut risque: 1
		Stade NP: 3		
				Glioblastome:2
				Stade NP : 1
**Traitement dans un autre centre** n = 6 (7, 8%)	0	Autre centre (Fes) : 3	0	0
		Privé : 3		
**Problème SE** n = 7 (9, 1%)	0	2	1	4 (Stade II)
**Progression/Altération** n= 5 (6, 5%)	0	5	0	0
**Refus du traitement**n = 4 (5, 2%)	0	0	0	4 (stade III)

## Discussion

L'observance est un enjeu majeur en médecine [1, 2]. La non-observance a un coût financier considérable pour les systèmes de soins. L'adhésion à un traitement ou à des recommandations thérapeutiques est le maillon principal de la chaîne entre l'intention de traiter et l'objectif de santé à atteindre. Les facteurs influençant la non-observance chez le malade cancéreux sont multiples; ils comprennent notamment les relations avec le personnel soignant, l'organisation pratique des soins et la perception qu'a le malade de sa maladie et de son traitement. L'impact du régime thérapeutique sur les habitudes de vie ainsi que les transformations physiques induites par le traitement sont également des obstacles importants à l'observance du traitement. La qualité des relations intrafamiliales joue aussi un rôle important [2]. Le problème de la concordance entre la prescription d'un traitement et sa prise effective n'est pas nouveau. Cependant, la confiance du milieu médical en l'autorité de ses propres directives l'a conduit à largement ignorer le problème de la non-observance jusqu’à dans les années 1970, moment de la redécouverte de ce concept. Depuis, la recherche a montré que la non-observance est un phénomène courant que l'on constate dans un grand nombre des pathologies, des plus bénignes aux plus sévères, et chez des patients de tous les âges [1, 3].

Une des principales difficultés concernant la recherche sur l'observance (compliance des AngloSaxons)est l'absence de consensus concernant sa définition. La plus couramment citée est le degré de coïncidence entre le comportement du patient (en termes de prise de médicaments, de suivi de régimes alimentaires, de modification du style de vie) et l'avis médical ou la recommandation de santé qui lui a été prescrite [3]. Bien que le terme d'observance soit encore le plus couramment trouvé dans la littérature médicale, son utilisation a souvent été remise en cause et remplacée par d'autres termes tel que l'adhésion ou concordance qui sont les principales alternatives qui ont été proposées. Ces termes reflètent des aspects différents de la question de l'adéquation entre la procédure thérapeutique proposée par une équipe soignante et ce que décide de faire le patient en pratique. De plus, l'utilisation de ces termes diffère selon les milieux (psychologie, sociologie, médecine) et selon les langues, constitue alors des difficultés supplémentaires [2]. L'analyse du vécu de la radiothérapie montre bien la nature des épreuves multiples auxquelles sont brutalement exposés les patients: épreuves d'un espace inconnu, complexe, générateur d'angoisse, d'une information souvent inadaptée, d'effets secondaires parfois trop banalisés, d'une solitude difficile à supporter tout au long du traitement avec un soutien parfois insuffisant de l’équipe soignante et de l'entourage familial, avec la crainte de l'abandon définitif en fin de traitement, d'une surveillance médicale jusqu'alors quotidienne et particulièrement rassurante ainsi que les longs retards dans le démarrage de radiothérapie qui ne sont manifestement pas bénéfiques pour nos patients, engendrant une détresse psychologique inévitable.

Une étude de l'OMS de juillet 2003 établit que la proportion de malades chroniques respectant leur traitement dans les pays développés n’était que de 50%, ce qui est en fait un problème grave et coûteux, car, (*l'observance insuffisante (..) entraîne des complications médicales et psychosociales, diminue la qualité de vie des patients, augmente la probabilité de développer des pharmaco-résistances et provoque un gaspillage des ressources* [4]). Si l'on entend par non-observance, le non-respect par le patient des recommandations et prescriptions thérapeutiques et sanitaires de l’équipe soignante, l'on peut se questionner sur l'ampleur de ce phénomène dans notre pays concernant une maladie nécessitant un traitement lourd et de longue durée comme le cancer qui a été bien moins explorée sur ce point que l'hypertension artérielle, l'asthme, le diabète ou la tuberculose [5, 6]. Or ce phénomène peut prendre différentes formes dans le parcours de soin d'un patient atteint du cancer: refus des traitements lourds (chimiothérapie, radiothérapie, hormonothérapie), non-respect des rendez-vous médicaux, des recommandations en soins de support (consultation psychologique, nutrition, traitement de la douleur), non prise des médicaments d'accompagnement qui luttent contre les effets secondaires (fatigue, nausées, troubles digestifs et intestinaux, douleurs articulaires et musculaires, irritations de la bouche et de la gorge, anémie), non-respect des consultations de surveillance en cours du traitement ou en période de rémission, refus du traitement en cas de rechute, mais aussi surconsommation des médicaments (la catégorie des trop observants).

Il est intéressant de relever que les différentes définitions de l'observance ont pour principe sous-jacent que la recommandation médicale du prescripteur est bonne ou qu'un comportement rationnel de la part du patient consiste à suivre précisément la prescription médicale [7]. Il a été suggéré que le concept même d'observance pourrait en fait être considéré comme fondamentalement anormal d'un point de vue moral et psychologique. En effet, une soumission aveugle et sans questionnement aux directives d'une personne pourrait être considérée comme un comportement plus déviant que la non-observance [8]. Bien qu'il ne se dégage pas de véritable consensus concernant les facteurs prédictifs de non-observance, il existe des liens possibles mis en évidence entre l'observance et certains facteurs socio-économiques, familiaux et liés à la maladie, ainsi que l'influence des relations patient-soignant. D'un point de vue statistique, certaines études laissent à désirer puisqu'un grand nombre des corrélations, entre l'observance et divers facteurs, mises en évidence sont faibles, mais l'existence des relations causales est néanmoins suggérée. De plus, la plupart de ces études sont transversales et non longitudinales, le terme de facteur prédictif n'est donc pas approprié (mais cependant fréquemment utilisé par les auteurs).

L'objectif de notre étude était de comprendre les différents aspects de la non-observance des malades suivis en radiothérapie. On a essayé de procéder par une enquête qualitative sur des échantillons restreints en explorant les questions suivantes: Quelles sont les raisons ou les motivations de ces formes de non-observance? Que se passe-t-il dans la tête de celui qui refuse, suit insuffisamment ou incorrectement son traitement: incompréhension, découragement, fatigue, déni de la maladie, expression de liberté, pulsion de mort? Revendique-t-il ou dissimule-t-il son attitude? Quelle est la part dans ce comportement:1)des facteurs socio-économiques: âge, sexe, catégorie socioprofessionnelle, éducation? 2) de la relation patient? équipes soignantes (confiance, communication), et patient? corps médical en général (image des médecins)? 3) de la pénibilité du traitement (nombre de séances, durée du traitement, nombre de médicaments, de prises, contraintes associées, effets secondaires multipliés)? 4) des représentations et croyances du patient sur la maladie et les médicaments? 5) du type de personnalité du patient: degré de confiance en soi, optimisme? 6) de l’évaluation du rapport coût / bénéfice du traitement par le patient et de son incidence sur le degré d'observance?

La présente étude a analysé l'impact du milieu socioculturel, des facteurs démographiques et ceux liés à la maladie sur le nombre des malades perdus de vue avant ou en cours du traitement par radiothérapie. Les patients plus âgés étaient plus susceptibles d’être perdus de vue. Cette catégorie est beaucoup plus dépendante des autres (soutien social pratique et émotionnel) et moins susceptible d’être demandeuse d'un traitement qu'elle considère source de douleur supplémentaire(certains aspects spécifiques du traitement comme la complexité du traitement, le nombre des séances, les éventuels effets secondaires, la durée du traitement, le coût) et qu'il n’évitera pas la mort (croyance religieuse). Vallikad et al. [9] a également observé que chez les patients qui étaient non-conformes au traitement, le manque du soutien interne, économique ainsi que le soulagement symptomatique a été parmi le raisonnement du patient pour arrêter le traitement. Sans un partenaire, le patient peut être moins susceptible de financer par ses propres moyens ses besoins médicaux ainsi que les besoins de sa famille en l'absence d'un chef de ménage. Les femmes mariées sont plus susceptibles d'assurer le suivi d'un traitement.

Dans la présente étude, l'analphabétisme a joué un rôle crucial dans l'augmentation des chances des patients perdus de vue (58% PDV étaient analphabètes). Les patients ayant fait des études primaires étaient plus susceptibles d’être perdus de vue par rapport aux patients ayant une éducation secondaire ou supérieure [10, 11]. Dans notre étude, les patients qui vivaient loin du centre de la radiothérapie étaient plus susceptibles d’être perdus de vue; 66% des PDV habitaient plus de 100 Km par rapport au centre de RTH. Les difficultés du transport peuvent être une des causes responsables de décourager les patients. Distance plus longue signifie une grande perte financière, l'accompagnant du patient doit prendre plus de temps hors du travail avec risque de perte du salaire, et aussi le coût des voyages et de l'hôtel après son arrivée au service de radiothérapie peut être coûteux pouvant décourager le patient à suivre son traitement [12]. Il importe d’évaluer et de distinguer les altérations physiologiques liées à l’âge, des co-morbidités vraies et du retentissement du cancer sur l’état général du patient. Les altérations physiologiques liées à l’âge, quoique inéluctables, sont très variables d'un patient à l'autre et d'une fonction à l'autre pour un même patient, volontiers difficiles à évaluer avec précision [13].

En revanche, les co-morbidités doivent être soigneusement recherchées avant l’établissement de la stratégie thérapeutique globale du cancer. En effet, une co-morbidité notable est retrouvée chez plus de 50% des patients atteints de cancer et âgés de plus de 60 ans. Dans notre série 94% de nos patients avaient des co-morbidités. Les résultats cancérologiques chez ces patients sont très mal connus, l’âge et l'existence des co-morbidités les excluant doublement des essais thérapeutiques. Si l'association âge plus co-morbidités fait volontiers récuser la chirurgie, certaines études montrent de façon surprenante que le recours à la radiothérapie serait lui aussi moins fréquent que pour des patients plus jeunes [13–15]. Les interactions entre radiothérapie et co-morbidités sont globalement de deux ordres: 1) la pathologie associée rend techniquement impossible la radiothérapie. C'est le cas des patients atteints de démence ou de maladie de Parkinson sévère pour lesquels l'immobilité requise pendant la séance est impossible. 2) la radiothérapie aggrave de façon inacceptable la pathologie associée. Ces situations sont très fréquentes. À titre d'exemple, on peut citer la radiothérapie thoracique chez les patients présentant une broncho-pneumopathie chronique obstructive sévère (BPCO). L'indice de performance est un facteur pronostique majeur en cancérologie, quel que soit l’âge du patient. Chez le patient jeune, il est en règle lié à l'extension et à la gravité de la maladie cancéreuse. Chez le sujet âgé ayant des pathologies associées, ses échelles (OMS, ECOG, Karnofsky) sont souvent mal adaptées car basées sur l'activité physique et la durée de l'alitement journalier. L’évaluation de l'indice de performance demande une attention particulière avant et pendant le traitement. Yamazaki et al. [16] ont observé une altération de l'indice de performance chez les sujets âgés (≥‘ 80 ans) traités par radiothérapie à visée palliative que chez ceux traités à visée curative, remettant en cause l'indication même de radiothérapie palliative chez ces patients. Les facteurs liés à la maladie influencent également les patients PDV. Dans la présente analyse, les patients avec des stades avancés de la maladie étaient plus susceptibles d’être perdus de vue, la majorité d'eux étaient stade III et IV (74%). Comme le patient devient plus dépendant, que la maladie progresse, le soutien familial devient également nécessaire. Ainsi, un patient aura besoin d'un soutien financier et physique et des ressources pour assurer un transport plus confortable. Divers facteurs socio-économiques tels que décrits ci-dessus s'ajoutent à la progression de la maladie et les mauvaises conditions sanitaires qui poussent le patient à ne pas faire son traitement [10, 17].

La deuxième analyse a montré que le revenu, le stade, la BPCO et la cardiopathie ischémique ont été associés à l'abandon du traitement. Le revenu a été évalué en fonction des divers facteurs (tels que l'occupation du patient, la profession du mari, s'ils louent ou possèdent leur maison), il est possible que les patients ont systématiquement sous-estimé leur revenu afin d’éviter de faire des paiements pour les traitements et les services de l'hôpital. Dans notre étude sept patients ont été perdus de vue à cause des problèmes socio-économiques. Les patients peuvent renoncer au traitement initial et les recommandations des médecins et de chercher une protection alternative lorsque les soins médicaux à un stade avancé semblent fournir très peu d'espoir et de soulagement. Une autre possibilité est que ces patients qui sont conscients de la progression de la maladie et peut-être dans le déni psychologique peuvent avoir recours à un autre moyen alternatif comme la thérapeutique spirituelle [12–14]. Concernant le stade de la maladie, il y a une possibilité d'accroître la souffrance par le traitement ainsi qu'une perte de la dignité de soi, de la détérioration physique sous l'effet de la tumeur et de la douleur et cela peut diminuer l'espérance du patient de poursuivre le traitement.

Le traitement par radiothérapie (curative ou palliative, schéma normo ou hypofractionné) doit être intégré à la stratégie thérapeutique globale qui peut comporter chirurgie, chimiothérapie, hormonothérapie ou thérapies ciblées. La prise en compte des co-morbidités et de l'indice de performance comme discutés ci-dessus s'adresse en fait au schéma thérapeutique dans son ensemble, en sachant que les effets indésirables des différents traitements s'additionnent, voire se potentialisent. Les soins de support doivent être envisagés d'emblée et réévalués lors de la consultation hebdomadaire en cours du traitement avec le radiothérapeute. La surveillance du poids et de l’état nutritionnel reste une priorité.

Les situations de refus, comme on a trouvé dans notre étude qui sont au nombre de quatre, sont pénibles et troublantes, tant pour les médecins et les équipes soignantes que pour les patients et leurs proches [18]. Elles ne sauraient être traitées ni par le rapport de force ni par leur acceptation passive. Elles posent des problèmes cliniques, relationnels et éthiques difficiles qui doivent être résolus dans le respect réciproque de l'autonomie, de la responsabilité et de la dignité. Ces situations illustrent aussi les relations complexes entre la société et sa médecine. Leur prévention ou leur résolution nécessitent d'abord des bonnes conditions de soin mais aussi la prise en compte des attentes et des craintes des patients, de leur psychologie, de leur situation sociale et familiale, de leurs références culturelles, idéologiques et éventuellement religieuses, des références législatives et des recommandations des sociétés savantes.

## Conclusion

La présente analyse a mis en évidence que les taux d’‘abandon sont associés à des facteurs liés à la maladie et qu’à la fois le patient et le médecin doivent être formés et être conscients de la façon dont les stades avancés de la maladie, le mauvais statut de performance ainsi que la combinaison des autres problèmes de santé peuvent suffisamment conduire le patient à l'abandon du traitement. Les patients et les médecins devraient être conscients de la puissance de l'espoir et les médecins devraient être plus conscients des besoins psychologiques des patients, de leur douleur et de leur désespoir, le médecin peut adapter les prescriptions thérapeutiques de la radiothérapie en fonction des besoins des patients afin de diminuer le taux d'abandon et de perte de vue et améliorer l'observance des malades atteints du cancer dans un proche avenir.
